# Special Issue “Molecular Mechanisms and Therapeutic Targets in Skin Diseases”

**DOI:** 10.3390/ijms27219677

**Published:** 2026-10-29

**Authors:** Hanna Myśliwiec

**Affiliations:** Department of Dermatology and Venereology, Medical University of Bialystok, 15-089 Bialystok, Poland; hanna.mysliwiec@umb.edu.pl

Skin diseases comprise a heterogeneous group of disorders in which genetic predisposition, immune dysregulation, environmental factors, metabolic alterations, and impaired epidermal homeostasis interact within complex molecular networks. Over the past decades, advances in molecular biology have substantially changed our understanding of their pathogenesis, revealing that clinically similar phenotypes may arise from distinct molecular pathways and that apparently skin-limited disorders may be associated with systemic inflammatory and metabolic alterations. These discoveries have also transformed therapeutic strategies in dermatology. The identification of disease-associated cytokines, signaling pathways, regulatory proteins, and epigenetic mechanisms has enabled the development of increasingly selective therapeutic approaches and has stimulated the search for biomarkers capable of predicting disease course and treatment response. Thus, contemporary dermatological research is progressively moving along a continuum from molecular mechanisms to biomarkers and targeted therapy, ultimately paving the way toward precision dermatology. The aim of this Special Issue, “Molecular Mechanisms and Therapeutic Targets in Skin Diseases”, was to highlight different aspects of this rapidly evolving field and to bring together studies addressing disease pathogenesis, potential biomarkers, and the molecular basis of therapeutic interventions.

Psoriasis provides a particularly illustrative example of how an expanding understanding of molecular mechanisms can reveal new dimensions of a complex systemic inflammatory disease. Beyond the well-established cytokine pathways, increasing attention is being directed toward less extensively explored regulatory proteins and metabolic processes that may contribute to the initiation and persistence of psoriatic inflammation. In this Special Issue, Matwiejuk et al. reviewed the potential roles of elafin, chemerin, and nicotinamide phosphoribosyltransferase (NAMPT/visfatin), highlighting their involvement in inflammation, immune regulation, metabolism, and epidermal homeostasis, as well as their potential relevance as biomarkers or therapeutic targets (contribution 1). Complementing this protein-centered perspective, Stepaniuk et al. investigated the kynurenine pathway of tryptophan metabolism in patients with psoriasis. Serum concentrations of tryptophan, kynurenic acid, and quinolinic acid were significantly higher in patients with psoriasis than in healthy controls, while urinary kynurenic acid/creatinine and quinolinic acid/creatinine ratios were also significantly increased (contribution 2). Moreover, the significant correlation between serum tryptophan and C-reactive protein (CRP) suggests a potential relationship between altered tryptophan metabolism and systemic inflammation and indicates that tryptophan may have potential value as an inflammatory marker (contribution 2). Whether these metabolic alterations primarily reflect chronic inflammation, contribute to psoriasis-associated systemic disturbances, or represent compensatory responses remains to be established. Together, these studies illustrate how the molecular landscape of psoriasis extends beyond classical immune mediators to encompass interconnected regulatory proteins and metabolic pathways, providing a broader framework for understanding disease heterogeneity and identifying potential novel biomarkers and therapeutic targets.

A broader understanding of skin disease also requires consideration of the spatial organization of molecular components within the skin and the complex interactions between multiple pathogenic pathways. Tokoro et al. used high-resolution mass spectrometry imaging to characterize the precise localization of skin lipids, demonstrating that different lipid species exhibit distinct spatial distributions across the skin layers (contribution 3). Their findings highlight the highly organized nature of cutaneous lipid metabolism and support the concept that the biological functions of individual lipids depend not only on their abundance but also on their site-specific localization. Importantly, as these analyses were performed in healthy skin, the resulting lipid localization data provide a valuable baseline, or healthy control dataset, for future mass spectrometry imaging (MSI)-based studies aimed at identifying disease-associated lipid alterations and potential lipid biomarkers (contribution 3). The complexity of molecular interactions underlying inflammatory dermatoses is further illustrated by the systematic review by Andrusiewicz et al., which summarized current evidence on the etiopathogenesis of rosacea (contribution 4). The authors highlighted the interplay between dysregulated innate immunity, neurovascular dysfunction, oxidative stress, and alterations in the skin microbiome, involving multiple mediators and signaling pathways, including pro-inflammatory cytokines, Toll-like receptors, NF-κB, MAPK, and STAT signaling (contribution 4). Together, these studies demonstrate that understanding skin disease increasingly requires moving beyond isolated molecular mediators toward integrated analyses of their spatial distribution and interactions within complex biological networks.

The growing understanding of molecular alterations in inflammatory skin diseases raises an important translational question: can this knowledge be converted into clinically useful biomarkers that help monitor disease activity or guide therapeutic decisions? Hołdrowicz et al. addressed this issue by analyzing and comparing the effects of methotrexate and biological therapies on the dynamics of selected blood-count-derived inflammatory indices in patients with psoriasis (contribution 5). Importantly, the study demonstrated that methotrexate and biologic treatment modify the dynamics of these inflammatory biomarkers in different ways, suggesting that changes in readily available hematological indices may reflect not only the reduction in systemic inflammation but also differences in the immunomodulatory effects of distinct therapeutic strategies (contribution 5). These findings support further investigation of blood-count-derived indices as simple and inexpensive tools for monitoring systemic inflammatory changes during psoriasis treatment. At a different level of biological complexity, Al-Sofi et al. systematically reviewed and meta-analyzed the association between genetic variants and response to biological therapies in chronic inflammatory diseases, including psoriasis, psoriatic arthritis, rheumatoid arthritis, and inflammatory bowel disease (contribution 6). Notably, genetic variants in TNFα, NLRP3, MYD88, and FcRγ were associated with response to TNF inhibitor therapy across several inflammatory diseases, supporting the potential relevance of shared genetic determinants of treatment response. Their work highlights the potential of genetic biomarkers to contribute to treatment-response prediction, while also demonstrating the current limitations of the evidence, including heterogeneity between studies and the need for further validation before genetic testing can be routinely implemented in clinical decision-making (contribution 6). Taken together, these studies illustrate two complementary directions in biomarker research—from readily accessible indicators reflecting treatment-associated changes in systemic inflammation to genetically determined predictors of therapeutic response—and underscore the potential value of integrating clinical, laboratory, and molecular information in the development of precision medicine.

Beyond their potential value as biomarkers, the identification of disease-associated molecular pathways provides a foundation for the development of novel targeted therapeutic strategies. Łyko et al. investigated the IL-36 cytokine family in pyoderma gangrenosum (PG), a rare neutrophilic dermatosis characterized by chronic and painful ulceration. The study revealed a dysregulated IL-36 cytokine profile in patients with PG, characterized by reduced serum levels of IL-36α and IL-36γ and increased levels of IL-36Ra, IL-37, and IL-38 compared with healthy controls (contribution 7). The authors suggested that this pattern may reflect a compensatory anti-inflammatory response to chronic inflammation. Moreover, the inverse correlation between IL-36α levels and ulcer size suggests a potential involvement of this cytokine in wound-healing processes. These findings further support the relevance of the IL-36 pathway in PG and provide a rationale for continued investigation of IL-36/IL-36R signaling as a potential therapeutic target. Another potentially relevant therapeutic pathway was explored by Song et al. in experimental models of atopic dermatitis (AD) (contribution 8). Using both cellular and animal models of AD-like inflammation, the authors demonstrated that *Sigesbeckia pubescens* Makino extract exerted anti-inflammatory effects associated with suppression of the JAK2/STAT signaling pathway (contribution 8). Treatment reduced the expression of inflammatory mediators and ameliorated AD-like skin inflammation, suggesting that modulation of JAK2/STAT signaling may contribute to its therapeutic effects (contribution 8). Together, these studies illustrate how identifying and experimentally characterizing disease-relevant inflammatory pathways can reveal potential molecular targets and provide a mechanistic basis for the development or evaluation of targeted therapeutic strategies.

The translation of molecular insights into therapeutic strategies is particularly evident in atopic dermatitis, where advances in understanding immune and epidermal signaling have led to increasingly targeted treatment approaches. Tsuji et al. provided new mechanistic insights into the effects of difamilast, a topical phosphodiesterase 4 (PDE4) inhibitor, demonstrating that it increased the production of soluble ST2 (sST2) in normal human epidermal keratinocytes through activation of the AHR–NRF2 axis (contribution 9). Difamilast induced the nuclear translocation of AHR followed by NRF2 activation, while knockdown experiments confirmed the involvement of both factors in difamilast-induced sST2 production (contribution 9). Acting as a decoy receptor, sST2 interfered with IL-33 binding to its membrane receptor ST2L and attenuated IL-33-induced basophil activation, including NF-κB and JNK phosphorylation and the upregulation of TNF-α, IL-5, and IL-13 (contribution 9). These findings identify the AHR–NRF2–sST2/IL-33 pathway as an additional molecular mechanism that may contribute to the therapeutic effects of difamilast in AD. Extending this mechanistic perspective to clinical treatment, Prados-Carmona et al. reviewed the rapidly evolving landscape of advanced systemic therapies for AD, linking current knowledge of disease immunopathogenesis with approved and emerging therapeutic strategies (contribution 10). Their review highlights how the identification of key components of type 2 inflammation and intracellular signaling pathways has enabled the development of monoclonal antibodies and JAK inhibitors, while additional molecular targets are being explored in clinical development (contribution 10). Together, these contributions illustrate the increasingly direct trajectory from the identification of disease-relevant molecular pathways to their pharmacological modulation and, ultimately, to more individualized therapeutic strategies for patients with AD.

Molecular approaches can also provide new insights into the biological effects of established therapeutic modalities. Okazaki et al. investigated the direct effects of 308 nm excimer light (EL-308) on the epidermis using a human organ-cultured skin model (contribution 11). EL-308 induced dose- and time-dependent changes in keratinocyte proliferation, apoptosis, and differentiation, while transcriptomic analysis revealed alterations in pathways related to metabolism, keratinization, and epidermal development (contribution 11). These findings provide new insights into the direct epidermal responses to EL-308 and demonstrate how molecular and ex vivo approaches can contribute to a better understanding of the biological effects of established dermatological treatments (contribution 11). Thus, this study complements the other contributions to this Special Issue by extending the continuum from molecular mechanisms and therapeutic targets to the molecular characterization of tissue responses to treatment.

Taken together, the contributions to this Special Issue illustrate the remarkable complexity of the molecular mechanisms underlying skin diseases and, at the same time, their growing translational relevance. Advances in the characterization of inflammatory mediators, metabolic pathways, lipid organization, and genetic determinants are expanding our understanding of disease pathogenesis and opening new opportunities for biomarker discovery. Importantly, these insights are increasingly being translated into the identification of therapeutic targets and a deeper understanding of the molecular effects of both novel and established treatments. Nevertheless, the clinical implementation of emerging biomarkers and molecular signatures will require further validation in larger and well-characterized patient populations, as well as integration with clinical and laboratory data. Ultimately, bridging molecular mechanisms, biomarkers, and targeted therapies may enable more precise patient stratification, improved prediction and monitoring of treatment response, and increasingly individualized therapeutic strategies. The studies collected in this Special Issue therefore reflect an ongoing transition from molecular mechanisms to biomarkers and targeted therapy, contributing to the continued development of precision dermatology.

